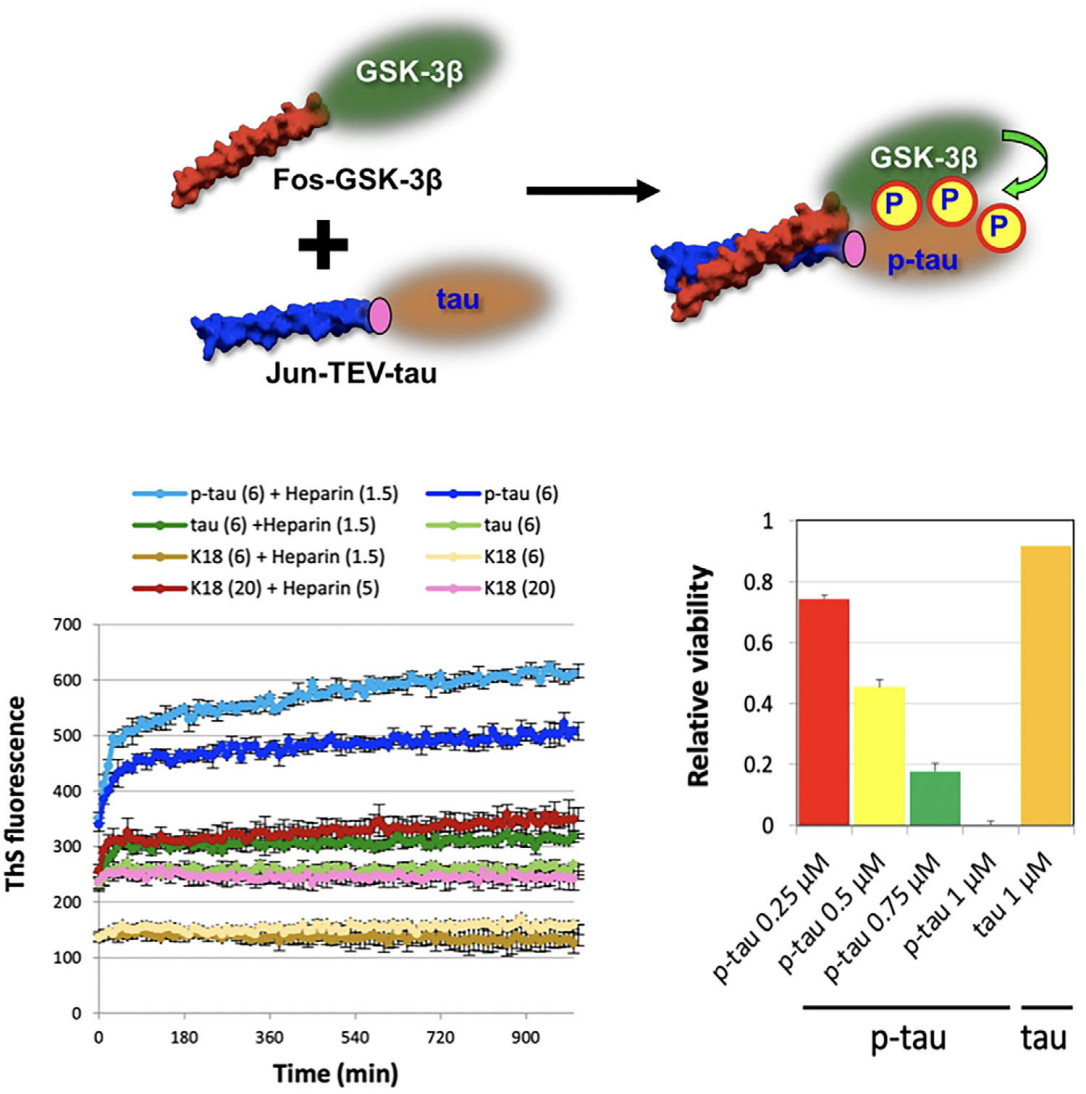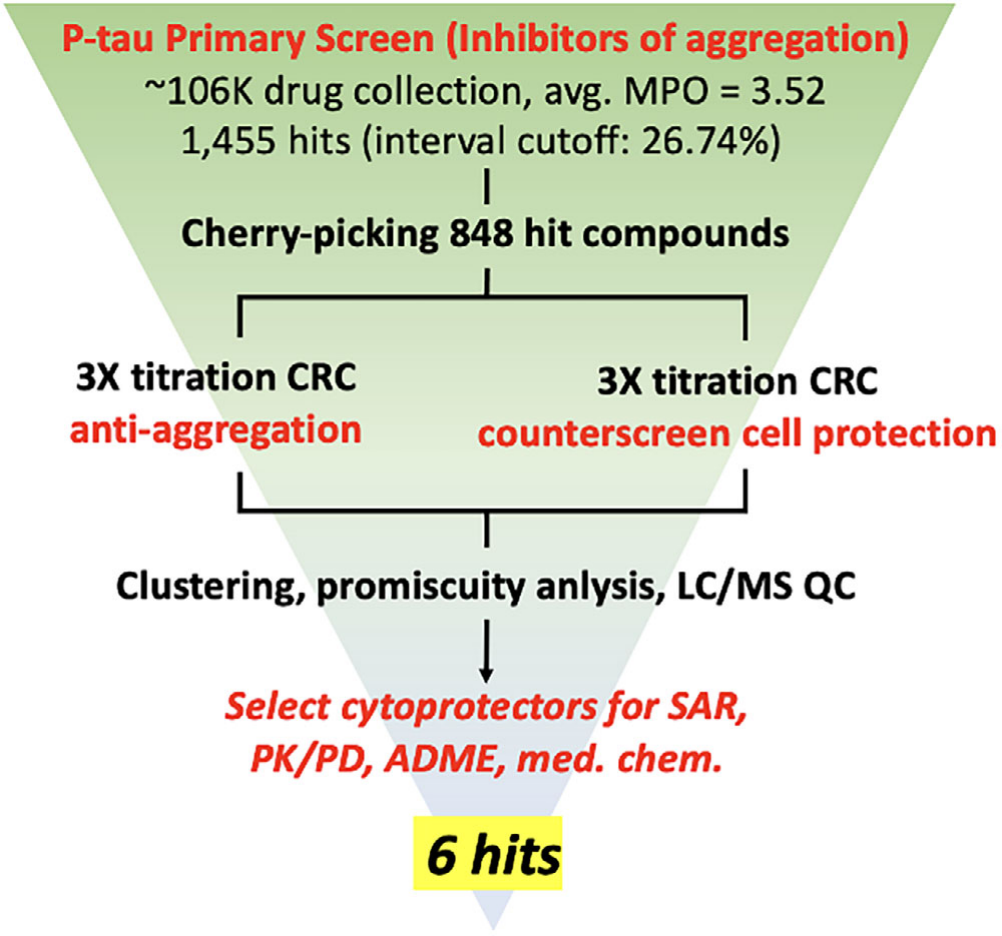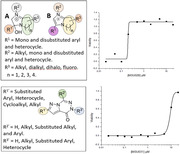# Alzheimer’s disease drug discovery centering on Hyperphosphorylated tau

**DOI:** 10.1002/alz.091367

**Published:** 2025-01-09

**Authors:** Kuang‐Wei Wang

**Affiliations:** ^1^ MSU, East Lansing, MI USA

## Abstract

**Background:**

Alzheimer’s Disease (AD) is a neurodegenerative disorder characterized by the accumulation of neurofibrillary tangles (NFTs) which consist primarily of hyperphosphorylated tau protein. Abnormal phosphorylated tau has been considered as a pathogenic species that impairs cellular function and propagates from neuron to neuron. AD affects millions of people around the world, however, there’s no effective drug that can prevent or cure the disease to date. Drug development based on A‐beta plague has encountered a bottleneck in the past few decades. Thus, finding a drug candidate against hyperphosphorylated tau pathology becomes a key topic.

**Method:**

With the use of recombinant hyperphosphorylated tau (p‐tau) which possesses several disease‐relevant characteristics, we have done a 100,000 – compound high‐throughput screening (HTS) based on p‐tau aggregation and cell‐based assays. The hits from the HTS were studied and modified with medicinal chemistry. The efficacy of synthetic compounds would be tested by our p‐tau cytotoxicity assay with SH‐SY5Y cells and then further verified with mice primary neurons and other functional assays.

**Result:**

From the high‐throughput screening, 29 hits were selected to be verified in our lab. We then focused on two potential compounds, MSU‐45028 (C9) and MSU‐45031 (C13), which showed overall better molecular, chemical, and biophysical characteristics. Within C9 and C13 series, a few analogs with improved structure‐activity relationship (SAR) property also performed good or even better EC_50_ against p‐tau cytotoxicity.

**Conclusion:**

The results showed that the hits identified from HTS could effectively inhibit p‐tau cytotoxicity. Our medicinal chemistry studies could improve the SAR while maintaining the efficacy of the compounds. The structural information of C9 and C13 analogs not only created potential AD drug candidates, but also provided hints for further drug development based on tau protein.